# Dopant-dependent structure–property relationships in functionalized graphene and MWCNTs for sustainable energy applications

**DOI:** 10.1038/s41598-026-63060-7

**Published:** 2026-07-23

**Authors:** Abdalrahman G. Al-Gamal, Walaa S. Gado, Muhammad A. Abo El-Khair, Asmaa S. Morshedy, Ahmed Mourtada Elseman, Khalid I. Kabel

**Affiliations:** 1 Petroleum Applications Department, Egyptian Petroleum Research Institute (EPRI), Nasr City, Cairo, 11727 Egypt; 2 Petrochemicals Department, Egyptian Petroleum Research Institute (EPRI), Nasr City, Cairo, 11727 Egypt; 3 Refinery Department, Egyptian Petroleum Research Institute (EPRI), Nasr City, Cairo, 11727 Egypt; 4 Electronic & Magnetic Materials Department, Advanced Materials Institute, Central Metallurgical Research and Development Institute (CMRDI), P.O. Box 87, Helwan, Cairo, 11421 Egypt

**Keywords:** Graphene halogen doping, Lithium-functionalized MWCNTs, Optical properties, Electrochemical impedance spectroscopy, Photoluminescence, Chemistry, Energy science and technology, Materials science, Nanoscience and technology

## Abstract

This study investigates the functionalization of carbon-based materials with halogen atoms (fluorine, bromine) and an alkali metal (lithium), subsequently examining the comparative impact of these modifications on their structural, optical, and electrical characteristics. *Graphene oxide* was modified with fluorine using hydrofluoric acid to produce fluorinated graphene (FG) and with bromide in the form of tetrabutylammonium bromide (TBAB) to produce G-TBAB. Separately, multi-walled carbon nanotubes (MWCNTs) were functionalized with lithium fluoride to form Li-MWCNTs. The materials were characterized via FTIR, XRD, SEM, TEM, AFM, and XPS. Optical properties were evaluated using UV–Vis and photoluminescence spectroscopy, and electrical behavior was assessed through electrochemical impedance spectroscopy (EIS). The findings demonstrate that among the three materials, FG exhibits the lowest charge transfer resistance (R_CT_ = 2.54 Ω.cm^−2^) and film resistance (R_f_ = 1.19 Ω.cm^−2^) in EIS measurements, suggesting more efficient charge transport under the tested conditions. G-TBAB and Li-MWCNTs show distinct EIS parameters, including higher capacitance values R_CT_ 12.91 Ω.cm^−2^ and 17.73 Ω.cm^−2^, which may be relevant for applications where charge accumulation is prioritized. These results indicate that hetero-functionalization of carbon allotropes provides a tunable platform, with each modification (F, Br, or Li) imparting distinct electrical and optical behavior.

## Introduction

Graphene, a two-dimensional monolayer of sp^2^-hybridized carbon atoms arranged in a honeycomb lattice, has revolutionized materials science since its isolation^1,2^. Its extraordinary properties-including ultra-high electron mobility (> 200,000 cm^2^ V⁻^1^ s⁻^1^), exceptional thermal conductivity, superior mechanical strength, and vast specific surface area-have positioned it as a cornerstone material for next-generation technologies in electronics, photonics, sensing, and energy conversion^3^. However, the absence of an intrinsic bandgap in pristine, defect-free graphene fundamentally limits its direct application in logic devices and certain optoelectronic systems that require a well-defined on/off switching behavior^4^. To address this limitation, extensive research efforts have focused on engineering graphene’s electronic structure. Among the various approaches, chemical doping has emerged as a highly effective and versatile strategy to precisely modulate graphene’s electrical conductivity, optical absorption, and surface reactivity^5,6^.

The introduction of heteroatoms or functional groups enables Fermi level tuning, bandgap opening, and improves interfacial compatibility, thereby tailoring graphene oxide for specific applications. Halogen doping, leveraging the high electronegativity and distinct bonding characteristics of elements such as fluorine and bromine, has attracted significant attention for inducing controllable electronic and optical modifications in graphene^7,8^. Fluorine, owing to its small atomic radius and extreme electronegativity, induces strong electron-withdrawing effects upon covalent bonding with carbon. This interaction locally transforms sp^2^ to sp^3^ hybridization, resulting in lattice distortion, bandgap opening, and, in many cases, enhanced photoluminescence (PL)^9,10^. In contrast, bromine doping predominantly introduces *p*-type behavior through charge-transfer interactions, while largely preserving the metallic nature of the graphene basal plane due to bromine’s lower electronegativity and larger van der Waals radius^11,12^. This fundamental contrast provides a powerful framework for rationally designing graphene derivatives with tunable electronic and optical properties via selective halogen functionalization.

In parallel with graphene research, carbon nanotubes (CNTs), particularly multi-walled carbon nanotubes (MWCNTs), have been extensively studied for their outstanding one-dimensional electrical conductivity, mechanical robustness, and high aspect ratio^13,14^. Moreover, functionalization of MWCNTs with alkali metals, particularly lithium, markedly augments their electrochemical performance by introducing additional charge carriers and modifying surface energetics. Lithium-functionalized MWCNTs (Li-MWCNTs) have demonstrated significant promise as high-capacity anode materials for lithium-ion batteries and as conductive additives in supercapacitors, owing to improved ion intercalation kinetics and charge storage capacity^15,16^.

Recent advances in composite solid electrolytes, such as dynamic crosslinked metal–organic framework/poly(ionic liquid) networks, highlight the critical importance of engineered interfaces and optimized ionic transport pathways in next-generation battery technologies^17^. This principle is equally applicable to the design of functionalized carbon electrodes. Despite notable progress in halogen-doped graphene and Li-functionalized CNTs individually, systematic studies exploring their combined effects within an integrated hybrid framework remain limited. The rational design of such multifunctional heterostructures offers the potential to overcome the intrinsic limitations of individual components, yielding composite materials with tunable conductivity, engineered band gaps, enhanced interfacial interactions, and superior mechanical and thermal performance.

The broader field of carbon-based hybrid materials has demonstrated the transformative impact of strategic material integration. For instance, heterostructures such as FeWO₄/g-C₃N₄^18^ and Z-scheme BiVO₄/g-C₃N₄/rGO^19^ have achieved exceptional solar-driven photocatalytic activity through optimized interfacial charge transfer. Similarly, nanocomposites such as AgFeO₂/g-C₃N₄/RGO^20^ and CuFe₂O₄/g-C₃N₄/rGO^21^ combine photocatalytic activity with magnetic separability and antibacterial functionality for wastewater treatment. Multifunctional architectures such as rGO/g-C₃N₄/FeTiO₃^22^ and ZnFe₂O₄/g-C₃N₄/rGO^23^ further illustrate how carbon-based platforms can deliver synergistic optoelectronic, catalytic, and biological performance. Collectively, these studies emphasize that the performance enhancement of carbon nanomaterials arises not only from doping but also from the deliberate creation of synergistic interfacial architectures.

The combination of halogen-doped graphene with Li-MWCNTs represents an innovative hybrid nanomaterial system with the potential to overcome limitations inherent in pristine graphene. Such a hybrid structure offers tunable electrical conductivity, a modifiable bandgap, enhanced interfacial interactions, and superior mechanical and thermal performance. Despite advances in graphene doping and CNT functionalization, studies exploring the combined effects of bromine and fluorine doping in graphene, coupled with lithium-functionalized MWCNTs, remain limited. Although Br- and F-doped graphene and Li-functionalized MWCNTs have individually attracted attention for their tunable electronic and optical properties, their comparative behavior within a unified hybrid framework remains insufficiently understood. In particular, the influence of dopant electronegativity and bonding characteristics on charge transport, recombination dynamics, and energy storage has not been systematically investigated. We hypothesize that the nature of the functionalizing species (F, Br, or Li) governs the balance between electrical conductivity, charge separation, and optical emission in carbon-based nanostructures. Specifically, fluorine doping is expected to enhance charge transport and suppress recombination due to its strong electron-withdrawing effect, whereas bromine and lithium functionalization are anticipated to promote charge storage and charge separation, respectively.

To evaluate this hypothesis, this study synthesizes and investigates three hetero-functionalized carbon allotropes: Br-doped graphene (G-TBAB), F-doped graphene (FG), and Li-functionalized multiwalled carbon nanotubes (Li-MWCNTs). Structural, optical, and electrochemical properties are systematically examined using FTIR, XPS, UV–Vis, photoluminescence (PL), and electrochemical impedance spectroscopy (EIS). The main objective is to establish structure–property relationships that clarify how different functionalization strategies modulate band structure, interfacial interactions, and charge-carrier dynamics, thereby identifying the most suitable material design for optoelectronic, energy-storage, and charge-balancing applications.

## Experimental section

### Materials

Graphite powder (Sigma-Aldrich, 99.99%), Tetrabutylammonium bromide (TBAB, Alfa Aesar, 99%), Hydrofluoric Acid (HF, 48%, Merck), Lithium Fluoride (LiF, Sigma-Aldrich, 99.99%). MWCNTs were supplied from the Egyptian Petroleum Research Institute (EPRI). These nanotubes have diameters ranging from 10 to 40 nm, lengths between 10 and 100 μm, and consist of approximately 40 to 50 walls. Ammonium hydroxide (NH_4_OH, 30%), sulfuric acid (H_2_SO_4_, 97%), Hydrochloric acid (HCl, 37%), Nitric acid (HNO_3_, 37%), hydrogen peroxide (H_2_O_2_, 10%), Ethanol, and Methanol were supplied from Honeywell Co. (USA). Indium tin oxide (ITO) (Sigma-Aldrich). Deionized water (18.2 MΩ cm). All chemicals were used as received.

### Synthesis of graphene oxide (GO)

GO was synthesized from graphite powder using an improved Hummers’ method^16^. Briefly, graphite (1 g) and NaNO_3_ (0.5 g) were dispersed in 23 ml of concentrated H₂SO₄ under continuous stirring in an ice bath to maintain a temperature below 5 °C. Subsequently, KMnO_4_ (3 g) was added gradually to prevent overheating. The mixture was stirred at 35 °C for 2 h, during which the suspension turned a dark brown color. Afterward, 46 ml of deionized water was slowly added, followed by 10 ml of H₂O₂ to terminate the reaction. The resulting solution was filtered and sequentially washed with 5% HCl and deionized water until a neutral pH was reached. The final graphene oxide was freeze-dried (lyophilized) to prevent aggregation and stored in a desiccator^15^.

### Functionalization of carbon materials

#### Synthesis of brominated *Graphene oxide* (G-TBAB)

A similar procedure involving the functionalization of graphene oxide (GO) with 1,3,5-triamino-2,4,6-trinitrobenzene (TATB) was employed to obtain noncovalently functionalized G-TBAB particles^2^. Briefly, (1 g) of GO was dispersed in 100 ml of distilled water (DI) and sonicated, followed by the gradual addition of (1 g) of TBAB. After 2 h of sonication, the resulting mixture was transferred to a Teflon-lined autoclave and heated at 200 °C for 12 h. The suspension was then allowed to settle for 24 h and subsequently centrifuged to collect the G-TBAB. The collected product was thoroughly washed with deionized water until the filtrate became clear, ensuring the removal of residual impurities, and finally dried at 80 °C.

#### Synthesis of fluorinated *Graphene oxide* (FG)

The fluorinated graphene (FG) was synthesized via a hydrothermal approach. Briefly, (1 g) of GO was suspended in a mixed solution of HF (40 wt%) and deionized water at a volume ratio of 10:90, followed by ultrasonication for 1 min to ensure homogeneous dispersion. The resulting mixture was transferred to a Teflon-lined autoclave and heated at 180 °C for 24 h. After naturally cooling to room temperature, the product was collected by filtration using a microporous membrane, thoroughly washed with ultrapure water to remove residual reactants, and freeze-dried to obtain FG^16^.

#### Synthesis of lithium-functionalized MWCNTs (Li-MWCNTs)

Lithium-functionalized multi-walled carbon nanotubes (MWCNTs) were synthesized via a solvent-mediated method in a Teflon-lined reactor. Briefly, (0.5 g) of MWCNTs was dispersed in 100 ml of a 1 M LiF aqueous solution and magnetically stirred for 24 h to enable lithium incorporation. The resulting solid product was collected by vacuum filtration, repeatedly washed with deionized water to remove unreacted species and residual salts, and subsequently dried under vacuum at 100 °C for 24 h ^24^.

#### Fabrication of thin films for electrical measurements

Thin films of each material (FG, G-TBAB, and Li-MWCNTs) were prepared by dispersing the powders in ethanol at a concentration of 1.0 mg/ml and ultrasonicating for 30 min to ensure homogeneous suspensions. Aliquots of 100 µl were drop-cast onto pre-cleaned ITO substrates (1 × 2 cm^2^) and dried at 60 °C to form uniform films. A gold counter electrode with a thickness of 50 nm was subsequently deposited by thermal evaporation through a shadow mask, yielding a two-electrode device configuration.

### Electrochemical impedance spectroscopy (EIS)

EIS measurements were performed using an Origalys (OrigaFlex 01A, potentiostat) in a two-electrode configuration, with the deposited carbon allotrope-based films on ITO serving as the working electrode and a gold (Au) wire as the counter electrode. The impedance spectra were recorded over a frequency range of 100 kHz to 250 mHz with a 150 mV AC amplitude. The experimental data were fitted to an electrical equivalent circuit (EEC) model using Origalyx software to extract key electrochemical parameters, including film resistance (R_f_), charge transfer resistance (R_CT_), constant phase element (CPE), Warburg impedance (W), and the CPE exponent (n). All measurements were performed in triplicate (n = 3), and the reported values represent the mean ± standard deviation. The CPE exponent (n) provides insight into the electrochemical behavior of the system, with (n = 0) resistive behavior, (n = 1) inductive behavior, (n = − 1) capacitive behavior, and (n = 0.5) Warburg impedance.

### Material characterization

The synthesized materials were characterized using a comprehensive suite of analytical techniques. Fourier-transform infrared (FTIR) spectroscopy (PerkinElmer Spectrum One) was used to identify functional groups over the wavenumber range of 400–4000 cm^−1^. Raman spectra were collected at room temperature using a 532 nm laser (1 µm spot size) on a Bruker, Germany, Senterra dispersive Raman spectrometer. Crystal structures and phase composition were analyzed by X-ray diffraction (XRD, PANalytical X’Pert PRO) using Cu Kα radiation (λ = 1.5406 Å). Surface morphology and elemental composition were examined by Field-emission scanning electron microscopy (FESEM, JEOL JSM-6360), coupled with energy-dispersive X-ray spectroscopy (EDX). High-resolution transmission electron microscopy (HR-TEM, JEOL JEM-2100F) was used to examine particle size, morphology and internal structure. Surface morphology and topographical features were examined by Atomic force microscopy (AFM) using a FLEX-AXIOM system operated at a vibration frequency of 154.7822 kHz. Optical properties were assessed using UV–Vis diffuse reflectance spectroscopy (JASCO V-770) to determine optical band gaps, while photoluminescence (PL) spectra were recorded using a JASCO FP-6500 spectrofluorometer with an excitation at 320 nm. X-ray photoelectron spectroscopy (XPS, Thermo Scientific K-Alpha) equipped with Al Kα source was utilized to analyze surface elemental composition and oxidation states.

## Results and discussion

### Structural and chemical characterization

#### FTIR analysis

**Fig. 1:**
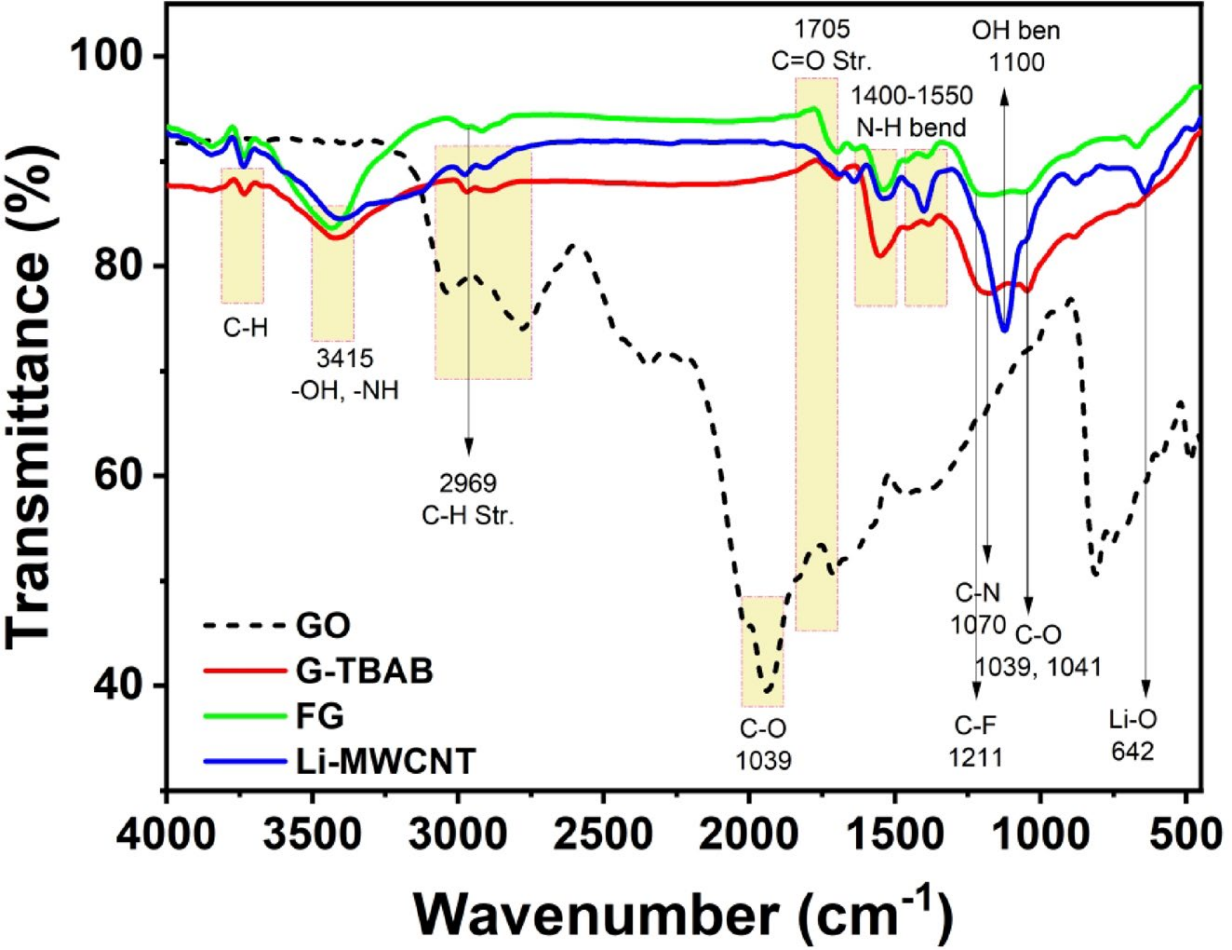
FTIR spectra of GO, G-TBAB, FG, and Li-MWCNTs.

The FTIR spectrum of graphene functionalized with TBAB exhibits distinct characteristic peaks, confirming successful noncovalent functionalization. The main absorption bands associated with graphene oxide (GO) appear at 3034 cm^−1^, 1712 cm^−1^, and 1039 cm^−1^, corresponding to C–H stretching, C=O stretching, and C–O stretching vibrations, respectively. However, the intensities of these bands are markedly reduced compared to pristine GO, as shown in Fig. 1^25^, indicating partial modification or masking of oxygen-containing functional groups upon TBAB incorporation. A broad absorption band at 3415 cm^−1^ is assigned to N–H stretching vibrations, characteristic of amine functionalities, suggesting the presence of TBAB or related surface interactions^26^. The peak at 2969 cm⁻^1^ represents C–H stretching vibrations, typical of aliphatic bonds and likely arising from the alkyl chains introduced by TBAB^27^. The band at 1554 cm^−1^ is attributed to N–H bending vibrations, further supporting the presence of amine groups associated with TBAB^28^. In addition, the absorption band at 1170 cm^−1^ is assigned to C–N stretching vibrations. The positively charged ammonium ion (R_4_N⁺) in TBAB can engage in electrostatic interactions with negatively charged oxygen-containing groups on GO, which likely contributes to the observed reduction in the intensities of epoxy, carboxyl, and hydroxyl-related peaks in the FTIR spectrum of the G-TBAB composite^29^. Collectively, these spectral features substantiate the successful attachment of TBAB onto the graphene surface through noncovalent interactions, as evidenced by the emergence of amine (N–H and C–N) vibrational modes and the attenuation of GO oxygenated functional-group signals. The FTIR spectrum of FG, green curve, exhibits characteristic absorption bands corresponding to C–O, C–F, C=C, C=O, and OH at 1041, 1211, 1541, 1705, and 3415 cm^−1^, respectively. The C–F stretching vibration, appearing in the range of 1200–1220 cm^−1^, is indicative of covalent bonding between fluorine atoms and *sp*^*3*^-hybridized carbon sites^30^. Notably, the intensities of the C–O and C=O stretching vibrations in FG are stronger than those typically observed in graphite fluoride, with a particularly pronounced enhancement of the C–O band relative to the C=O band. This observation suggests that oxygen-containing functional groups in FG are predominantly incorporated as C–O linkages^31^. The FTIR spectrum for Li-MWCNTs displays absorption bands at approximately 2973.76 and 2890.41 cm^−1^, which are attributed to C–H stretching vibrations. The bands observed near 1640.01 and 1540.07 cm^−1^ correspond to the C=O and C=C stretching vibrations, respectively. A distinct absorption peak at around 642.89 cm^−1^ is assigned to Li–O vibrations, indicating the formation of lithium-oxygen bonds following the doping process^32^. The relatively strong intensity of this band suggests substantial Li incorporation within the MWCNTs structure^24^. Additional absorption bands at 1401.70, 1134.62, and 3402.97 cm^−1^ are attributed to C–O stretching, hydroxyl bending, and OH stretching vibrations of adsorbed water molecules, respectively, further confirming the successful functionalization of the material^33,34^.

#### Raman analysis

**Fig. 2:**
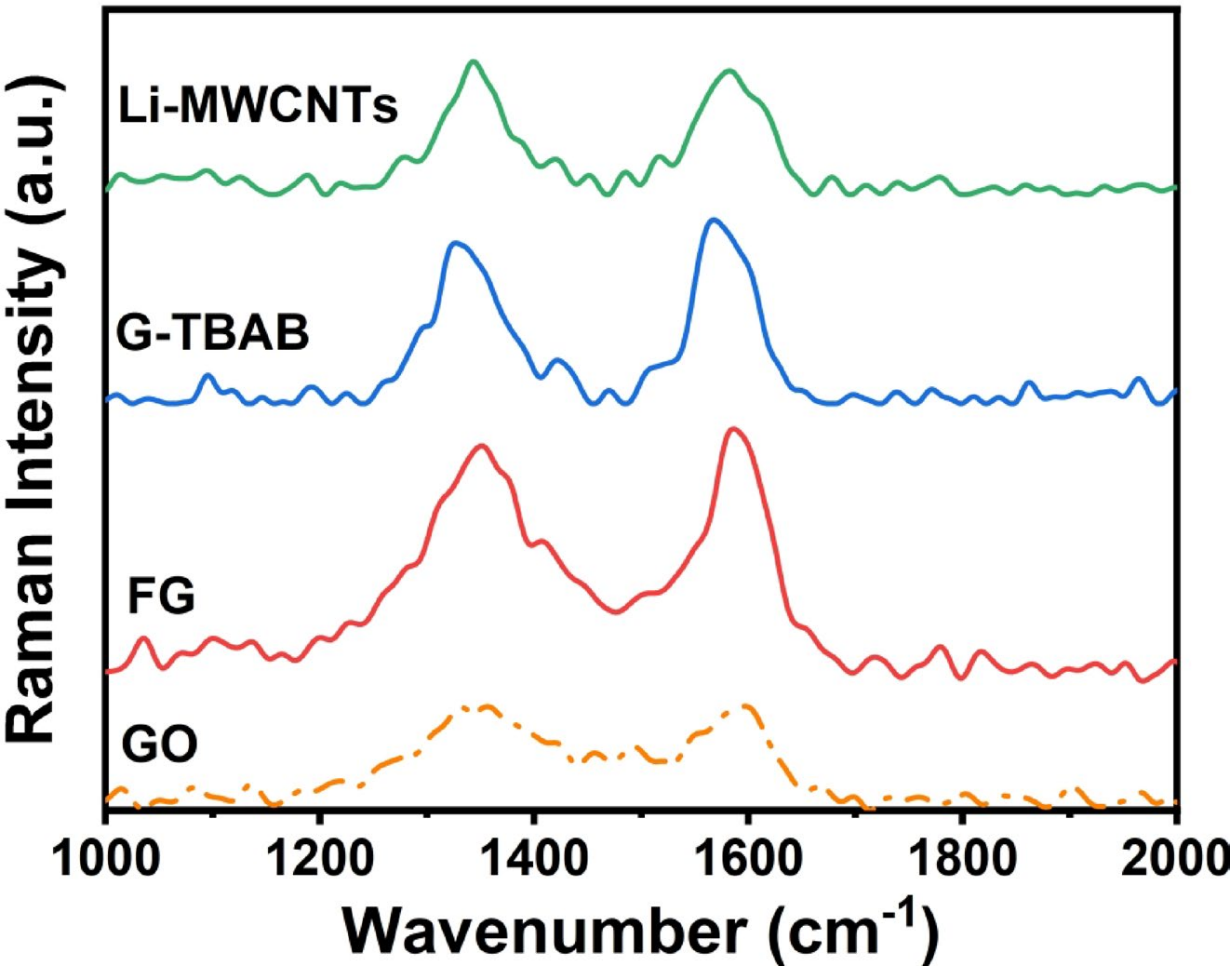
Raman spectra of GO, G-TBAB, FG, and Li-MWCNTs.

Raman spectroscopy was used to evaluate the structural integrity and defect density of the functionalized GO derivatives. The (I_D_/I_G_) ratio was calculated from the Raman spectra (Fig. 2) to assess the degree of structural disorder. The obtained I_D_/I_G_ values were 0.998 for GO, 0.933 for FG, 0.874 for G-TBAB, and 1.074 for Li-MWCNTs. Among the samples, G-TBAB exhibited the lowest I_D_/I_G_ ratio and a blue-shifted G band, indicating partial restoration of the sp^2^ carbon network via GO reduction, consistent with its improved electrical conductivity. In contrast, Li-MWCNTs showed the highest I_D_/I_G_ ratio and a broadened G band (FWHM ≈ 80 cm⁻^1^), suggesting increased structural disorder and defect formation induced by doping. These defects can provide additional active sites for charge storage but may also increase charge-transfer resistance. FG exhibited intermediate characteristics, indicating a balance between defect healing and defect generation. Overall, the relationship between the I_D_/I_G_ ratio and electrochemical performance highlights the importance of defect engineering: reduced disorder favors electrical conductivity, while controlled defect introduction can enhance capacitive charge storage.

#### X-ray diffraction (XRD) analysis

**Fig. 3:**
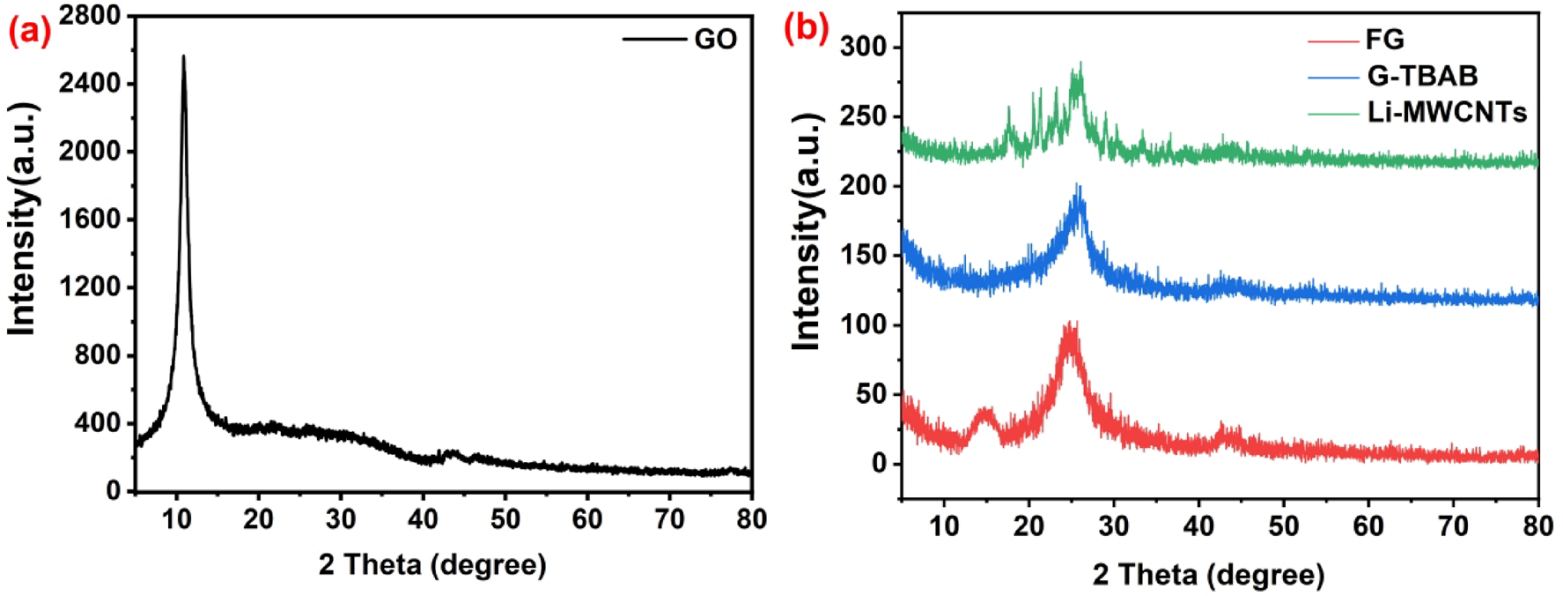
XRD patterns of (**a**) GO. (**b**) G-TBAB, FG, and Li-MWCNTs.

XRD was employed to evaluate the crystallographic modifications induced by functionalization. Following the oxidation and exfoliation of graphite via sonication, GO was formed, exhibiting a characteristic diffraction peak to a low reflection angle of 10.78º, which is associated with the presence of oxygen-rich functional groups, as shown in Fig. 3. The XRD pattern of TBAB-functionalized GO (Fig. 3) shows a pronounced structural transformation, evidenced by the disappearance of the characteristic peak at ~ 10°, corresponding to oxygenated functional groups (e.g., OH, C–O–C, and COOH). In contrast, a new diffraction peak appears at 24.8°, indexed to the (311) plane, indicating significant structural reorganization induced by TBAB functionalization^35^. As shown in Fig. 3, hydrothermal treatment of the GO dispersion with HF at 180 °C for 30 h results in the disappearance of the GO peak at 10.8° and the emergence of a broad diffraction peak centered at approximately 24.7°, corresponding to an interlayer spacing of 0.52 nm. This shift indicates effective removal of oxygen-containing functional groups and the partial restoration of the graphene framework. Upon further addition of HF, the resulting fluorinated graphene exhibits an additional diffraction peak at approximately 15.1°, with an interlayer spacing of 0.72 nm. This peak, assigned to the (001) plane of a hexagonal lattice, reflects a high degree of fluorine incorporation within the graphene structure^16,36^. The XRD pattern of Li-functionalized MWCNTs (Fig. 3) displays a prominent diffraction peak at 25.58°, corresponding to the (002) plane of MWCNTs, which is characteristic of the graphitic structure. Following LiF functionalization, additional diffraction peaks are observed at 17.1°, 36.2°, 43.2°, and 69.3°, which can be indexed to the characteristic reflections of LiF. The appearance of these peaks confirms the successful incorporation of LiF into the MWCNT framework and indicates enhanced crystallinity of the functionalized material^37,38^.

#### XPS analysis

**Fig. 4:**
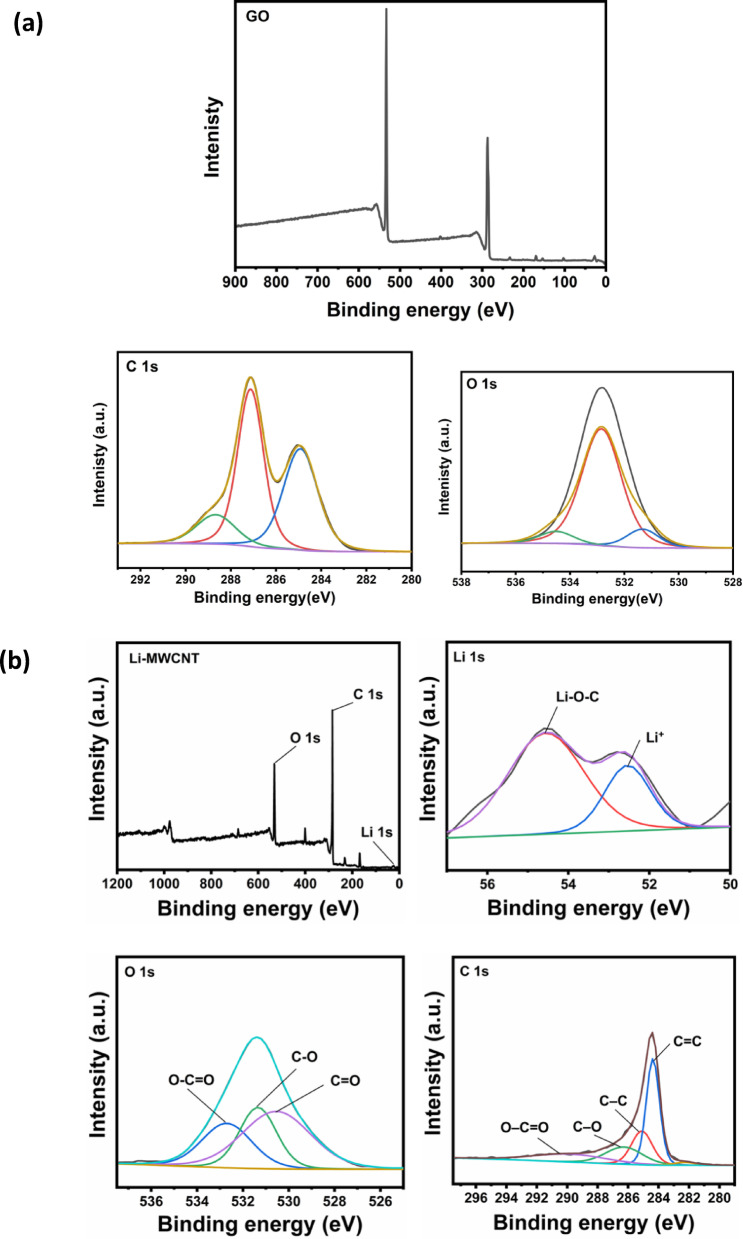

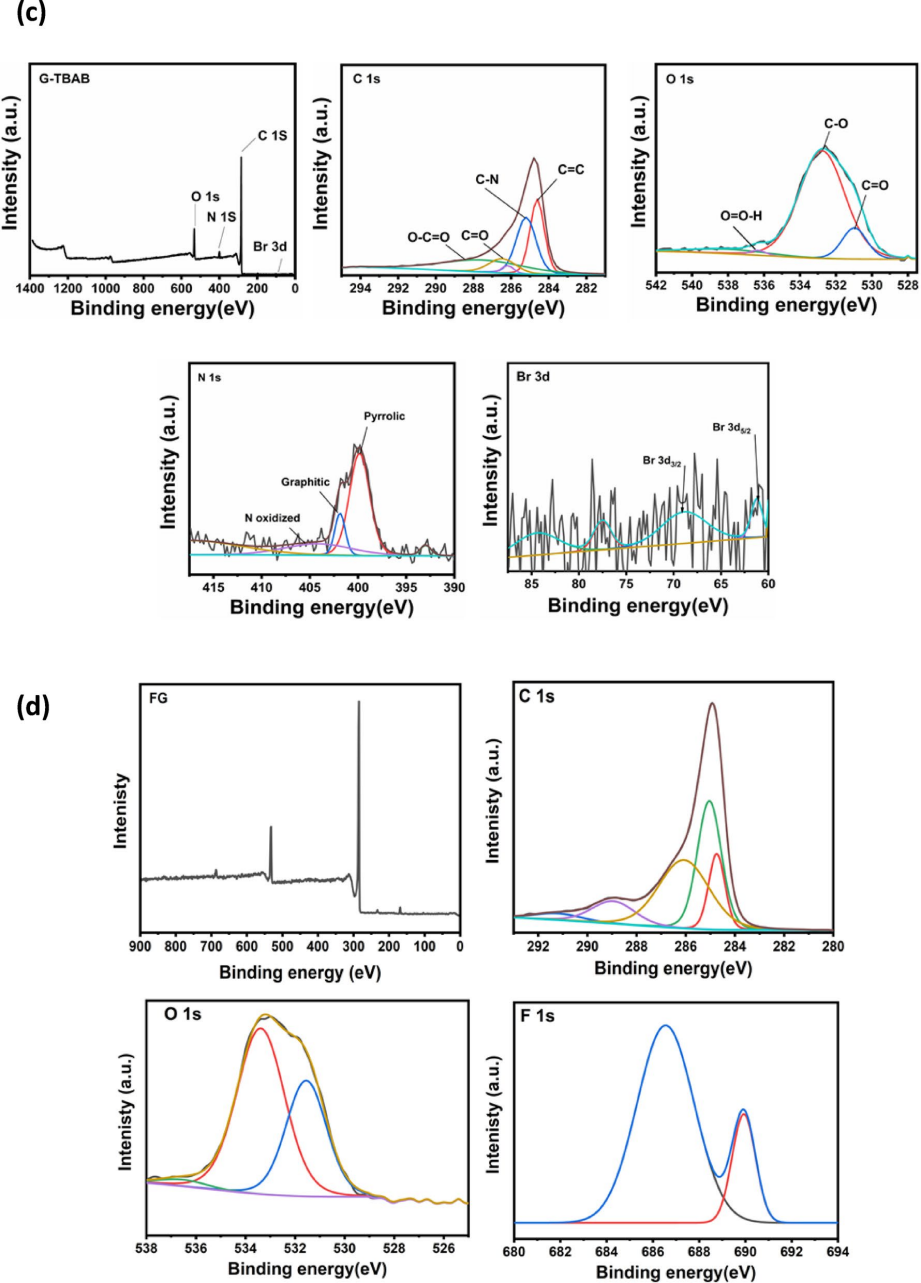
XPS survey and high-resolution spectra of (**a**) GO, (**b**) Li-MWCNTs, (**c**) G-TBAB, and (**d**) FG, confirming the successful incorporation of the corresponding dopants and the changes in surface elemental composition after functionalization.

XPS was conducted to investigate the surface composition and chemical states of Li-MWCNTs, G-TBAB, and FG. For Li-MWCNTs (Fig. 4b), the survey spectrum confirms the presence of Li, C, and O. The high-resolution Li 1 s spectrum exhibits two distinct peaks at approximately 52.5 eV and 55.0 eV, which are attributed to Li^+^ species associated with Li–O bonding and more highly oxidized lithium environments, respectively. The O 1 s spectrum displays three components at 530.5 eV (C=O), 531.4 eV (C–O), and 532.8 eV (O–C=O), indicating the presence of multiple oxygen-containing functional groups that facilitate lithium anchoring on the MWCNT surface^39^. The C 1 s spectrum reveals characteristic peaks at 284.5 eV (*sp*^*2*^ C=C), 285.5 eV (*sp*^*3*^ C–C/C–H), 286.3 eV (C–O), and 290.5 eV (O–C=O), confirming preservation of the graphitic framework alongside surface functionalization^40^. For G-TBAB (Fig. 4c), the survey spectrum confirms the presence of C, O, N, and Br, indicating successful incorporation of TBAB. The high-resolution C 1 s spectrum shows peaks at 284.6 eV (C=C), 285.2 eV (C–N), 286.5 eV (C=O), and 288.3 eV (O–C=O), consistent with nitrogen-containing functional groups introduced during functionalization. The O 1 s region exhibits components at 531.0 eV (C=O), 532.6 eV (C–O), and 536.4 eV (O–OH)^41^. The N 1 s spectrum reveals contributions from pyrolytic, graphitic, and oxidized nitrogen species at binding energies of 399.8, 401.9, and 405.4 eV, respectively^42^. In addition, the Br 3d spectrum confirms bromine incorporation, displaying characteristic doublet peaks at 61.2 eV (3d_5/2_) and 68.9 eV (3d_3/2_), along with higher-binding-energy features. For FG (Fig. 4d), the survey spectrum confirms the presence of C, O, N, and F. The O 1 s spectrum exhibits peaks at 531.3 eV (C=O), 533.1 eV (C–O), and 535.6 eV (O–C=O). The N 1 s spectrum shows four components at 395.5 eV (pyrrolic N), 398.6 eV (pyridinic N), 399.9 eV (aminic N), and 401.2 eV (graphitic N), indicating nitrogen incorporated in multiple chemical environments^27^. The high-resolution C 1 s spectrum displays peaks at 284.4 eV (*sp*^*2*^ C=C), 284.9 eV (*sp*^*3*^ C–C), 285.8 eV (C–N/C–O), and 288.0 eV (C=O), confirming the structural integrity of the graphene lattice alongside the presence of oxygen- and nitrogen-containing functional groups^39^. According to quantitative XPS analysis of all modified GO samples (Table 1), the dopants significantly modified the electronic structure of the carbon framework. FG contained 1.35 at.% F and exhibited the lowest C/O ratio (6.88), indicating electron withdrawal from the carbon network by highly electronegative fluorine atoms. G-TBAB showed a higher C/O ratio (9.03) with the presence of N (2.87 at.%) and Br (0.33 at.%), suggesting enhanced restoration of conjugated carbon domains through charge-transfer interactions. Li-MWCNTs exhibited the highest C/O ratio (9.19), while the presence of Li (0.08 at.%) indicates electron donation to the carbon matrix and modification of the local electronic environment. These results demonstrate that the nature of the dopant governs the electronic structure through electron-withdrawing (F), charge-transfer (Br/N), and electron-donating (Li) effects.

**Table 1:** XPS elemental composition (at.%), dopant content (F, Br, N, and Li), and C/O ratios of FG, G-TBAB, and Li-MWCNTs.

Sample	C (%)	O (%)	Dopant (%)	C/O
GO	64.53	64.53	–	1.82
FG	86.14	12.51	F = 1.35	6.88
G-TBAB	87.15	9.65	N = 2.87, Br = 0.33	9.03
Li-MWCNTs	90.12	9.80	Li = 0.08	9.19

### Morphological and structural analysis

#### Scanning electron microscopy (SEM)

**Fig. 5:**
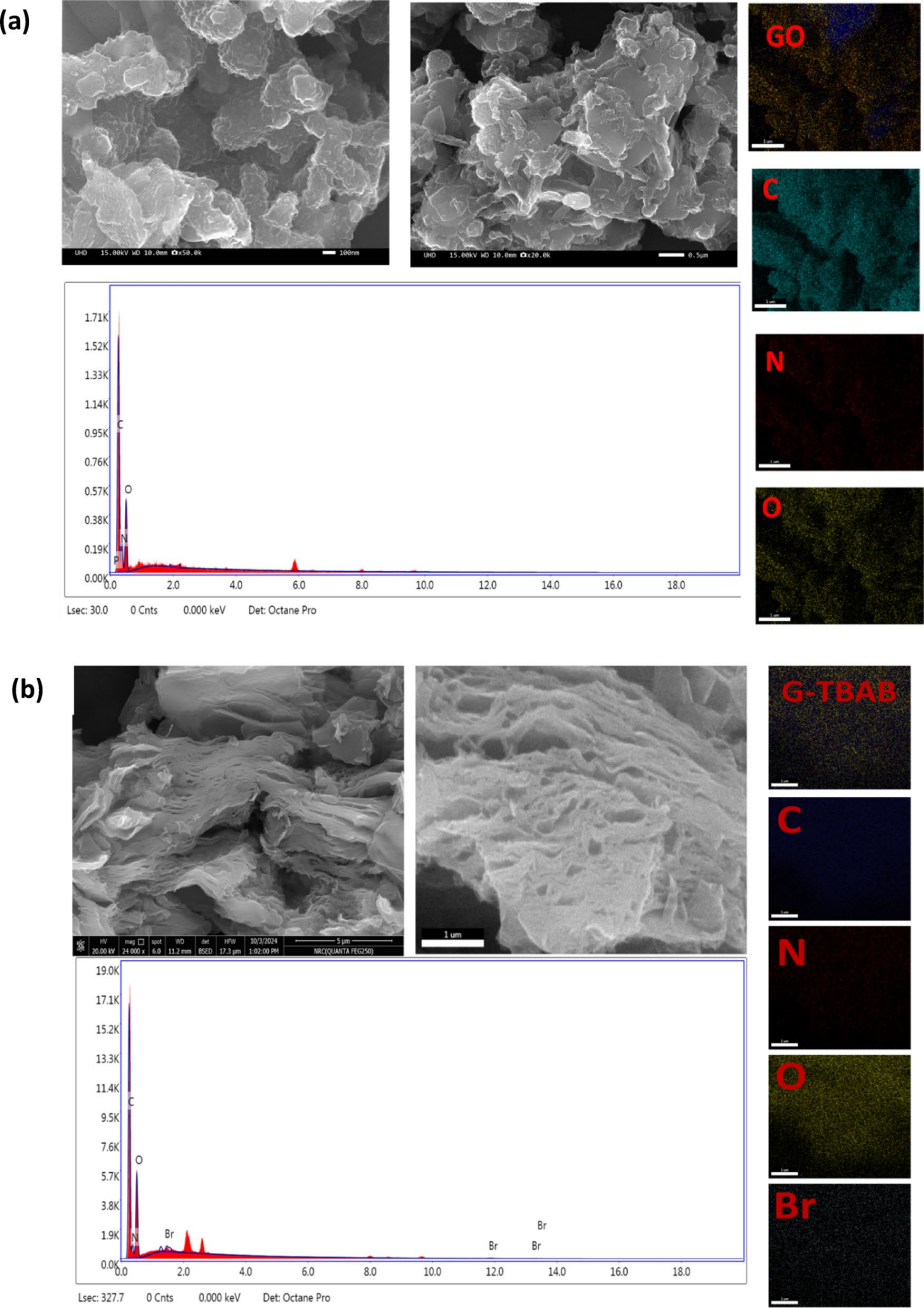

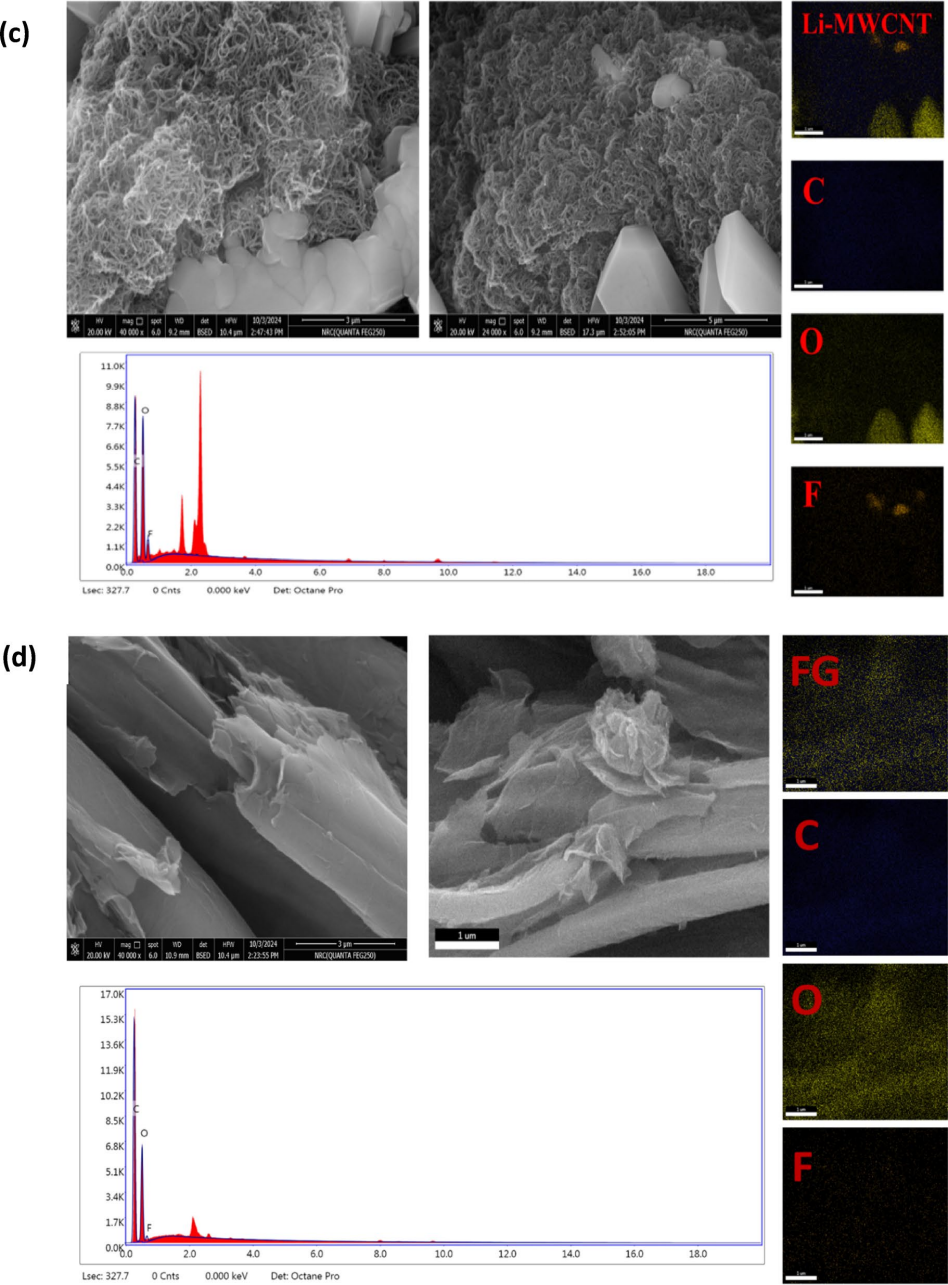
SEM micrographs and EDX of (**a**) GO, (**b**) G-TBAB, (**c**) Li-MWCNTs, and (**d**) FG illustrate the morphological changes induced by fluorine, TBAB, and Li functionalization. EDX elemental mapping confirms the successful incorporation of the corresponding dopants.

Scanning electron microscopy (SEM) revealed distinct morphological differences among the functionalized materials, as shown in Fig. 5. The G-TBAB sample exhibits a flake-like morphology with reduced aggregation, which can be attributed to steric hindrance induced by tetrabutylammonium groups. In contrast, fluorinated graphene (FG) exhibits a more compact layered structure, consistent with increased structural rigidity due to fluorine incorporation. Li-MWCNTs retain their characteristic tubular morphology, with LiF crystallites uniformly distributed on the outer surfaces of the nanotubes. SEM images of GO reveal irregular, wrinkled, and stacked sheet-like flake morphology, characteristic of GO, as shown in Fig. 5a. The SEM–EDX spectrum confirms that the sample is primarily composed of carbon and oxygen, with detectable impurities introduced during preparation. As shown in Fig. 5b, G-TBAB exhibits a layered architecture interspersed with flake-like features, suggesting partial exfoliation and separation of graphene sheets. The presence of bulky tetrabutylammonium moieties likely inhibits restacking by introducing steric barriers between adjacent layers. EDX mapping further confirms successful functionalization by detecting both nitrogen and bromine. The partially retained layered morphology may promote efficient electron-transport pathways, thereby enhancing the suitability of G-TBAB for electrochemical applications. The SEM images of Li-MWCNTs (Fig. 5c) reveal an interconnected network of entangled nanotubes with smooth surfaces, characteristic of MWCNT assemblies. The nanotubes appear as dense bundles, with no noticeable structural damage or fragmentation, indicating that the functionalization process preserves the MWCNTs’ structural integrity. EDX mapping confirms the presence of carbon, oxygen, and fluorine. However, lithium is not detected due to its low atomic number and the inherent limitations of EDX for detecting light elements. SEM analysis of FG (Fig. 5d) reveals a sheet-like morphology with well-defined flake characteristics, indicative of exfoliated graphene layers. EDX spectra confirms the presence of fluorine, providing direct evidence of successful fluorine doping within the graphene framework.

#### Transmission electron microscopy (TEM)

TEM analysis reveals distinct structural features of GO and its functionalized derivatives. GO exhibit disordered, wrinkled, and folded sheet-like morphologies with an expanded interlayer spacing of approximately 0.84 nm, arising from the introduction of oxygen-containing functional groups that disrupt the planner *sp*^*2*^ carbon lattice. In contrast, FG exhibits comparatively flatter, more rigid sheets with localized lattice distortion and a reduced interlayer spacing of approximately 0.36 nm, confirming partial fluorination without extensive structural damage. Similarly, G-TBAB presents well-dispersed flake-like structures with an interlayer spacing of ~ 0.36 nm, indicative of reduced aggregation and partial restoration of graphene stacking. Li-MWCNTs retain their characteristic tubular morphology, with localized surface roughness attributed to LiF incorporation, while preserving the integrity of the nanotube core.

**Fig. 6:**
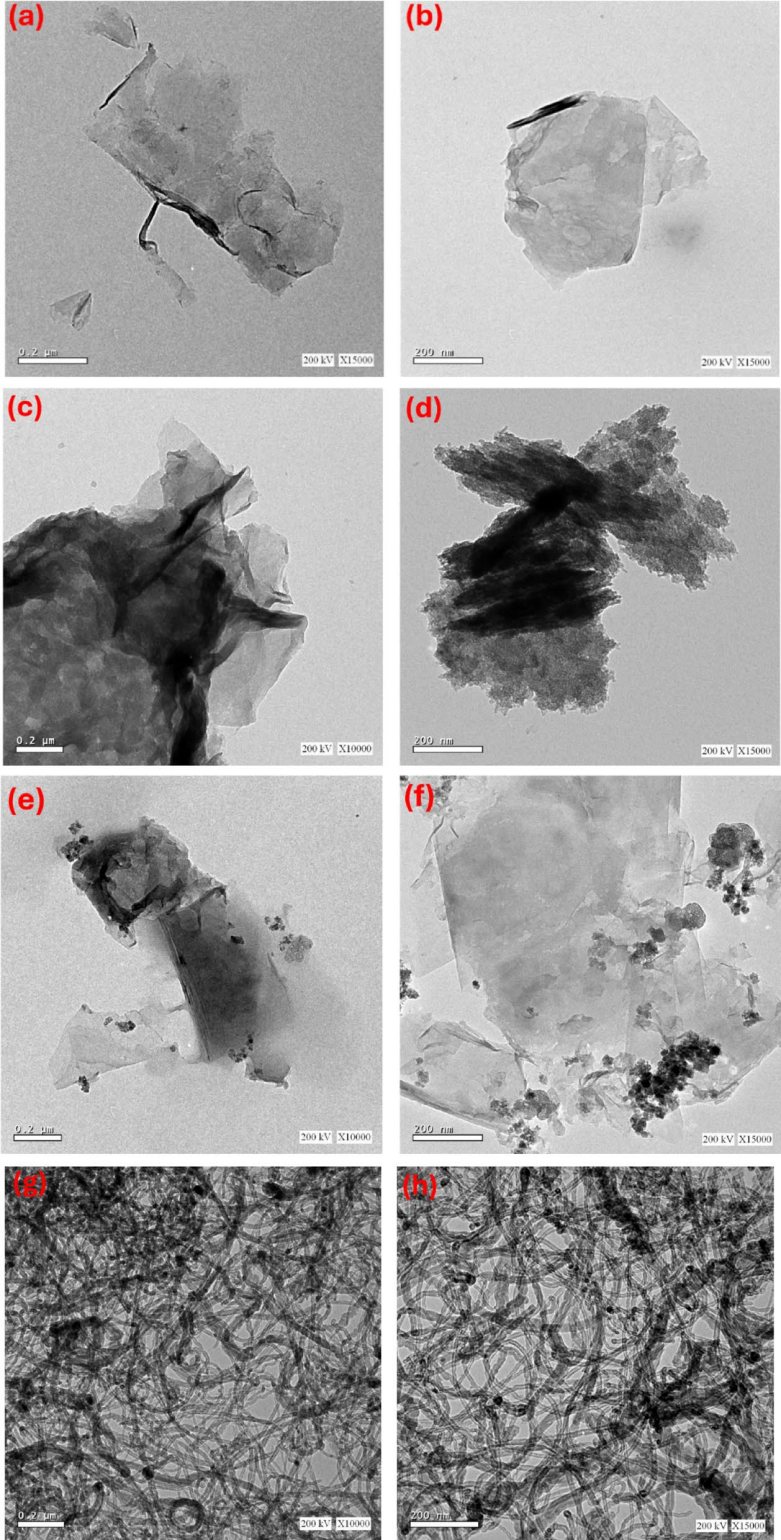
HR-TEM of GO (**a** and **b**), FG (**c** and **d**), G-TBAB (**e** and **f**), and Li-MWCNTs (**g** and **h**) at two different magnifications.

Figure 6 presents high-resolution TEM (HR-TEM) images of GO and the functionalized carbon-based derivatives. As shown in Fig. 6a and b, GO exhibits wrinkling and folding, accompanied by an amorphous region, resulting from the presence of hydroxyl, epoxy, and carboxyl groups. These oxygen functionalities expand the interlayer spacing to approximately 0.84 nm, compared to ~ 0.34 nm in pristine graphene, and introduce a high density of structural defects that disrupt the long-range *sp*^*2*^ ordering. In Comparison, the HR-TEM images of FG (Fig. 6c and d), reveal a more ordered and layered morphology, reflecting enhanced structure rigidity induced by fluorine incorporation. The strong electronegativity of fluorine stabilizes the graphene layers and suppresses excessive curling. The observed lattice spacing of − 0.35–0.37 nm indicates low-to-moderate fluorine doping, accompanied by minor, localized defects, while largely preserving the graphene lattice. This controlled functionalization enhances thermal stability, chemical resistance, and surface energy, rendering FG attractive for applications such as supercapacitors and solid lubrication. The G-TBAB sample (Fig. 6e and f) exhibits a dispersed, flake-like morphology with significantly reduced aggregation. The interlayer spacing ~ 0.36 nm suggests partial recovery of the graphene framework following functionalization. The relatively uniform attachments of TBAB molecules contribute to improved dispersion and fewer large-scale defects. However, the noncovalent nature of TBAB-graphene interactions may result in weaker interfacial stability, potentially limiting long-term durability under harsh operating conditions. Finally, Li-MWCNTs (Fig. 6g and h) display a well-defined tubular morphology with concentric graphitic layers. The interlayer spacing remains approximately 0.34 nm, while the outer walls exhibit slight expansion due to LiF intercalation and surface interactions. Localized structural disorder appears near the doped region; however, the nanotube cores remain largely intact, preserving their intrinsic mechanical strength and electrical conductivity. This combination of structural integrity and localized lithium functionalization highlights the potential of Li-MWCNTs for advanced electrochemical energy storage and electronic applications. A comparative summary of the structural characteristics of GO and its functionalization derivatives, including surface morphology, interlayer spacing, defect density, and associated electronic properties, is provided in Table 2.

**Table 2:** HR-TEM features of GO, FG, G-TBAB, and Li-MWCNTs.

Feature	GO	FG	G-TBAB	Li-MWCNTs
Morphology	Wrinkled, disordered	Rigid, layered	Dispersed, flake-like	Tubular, concentric
Lattice spacing (nm)	− 0.84	− 0.35–0.37	− 0.36	~ 0.34 (inner), ~ 0.36 (outer)
Defects	High (oxidation defects)	Scattered (fluorine)	Few-layered, fewer defects	Localized (outer walls)
Structural integrity	Disrupted	Well-maintained	Stable and well-dispersed	Strong core, local defects
Functional impact	Insulating	Improved thermal stability	Improved solubility	Enhanced electrochemical properties

#### AFM characterizations

**Fig. 7:**
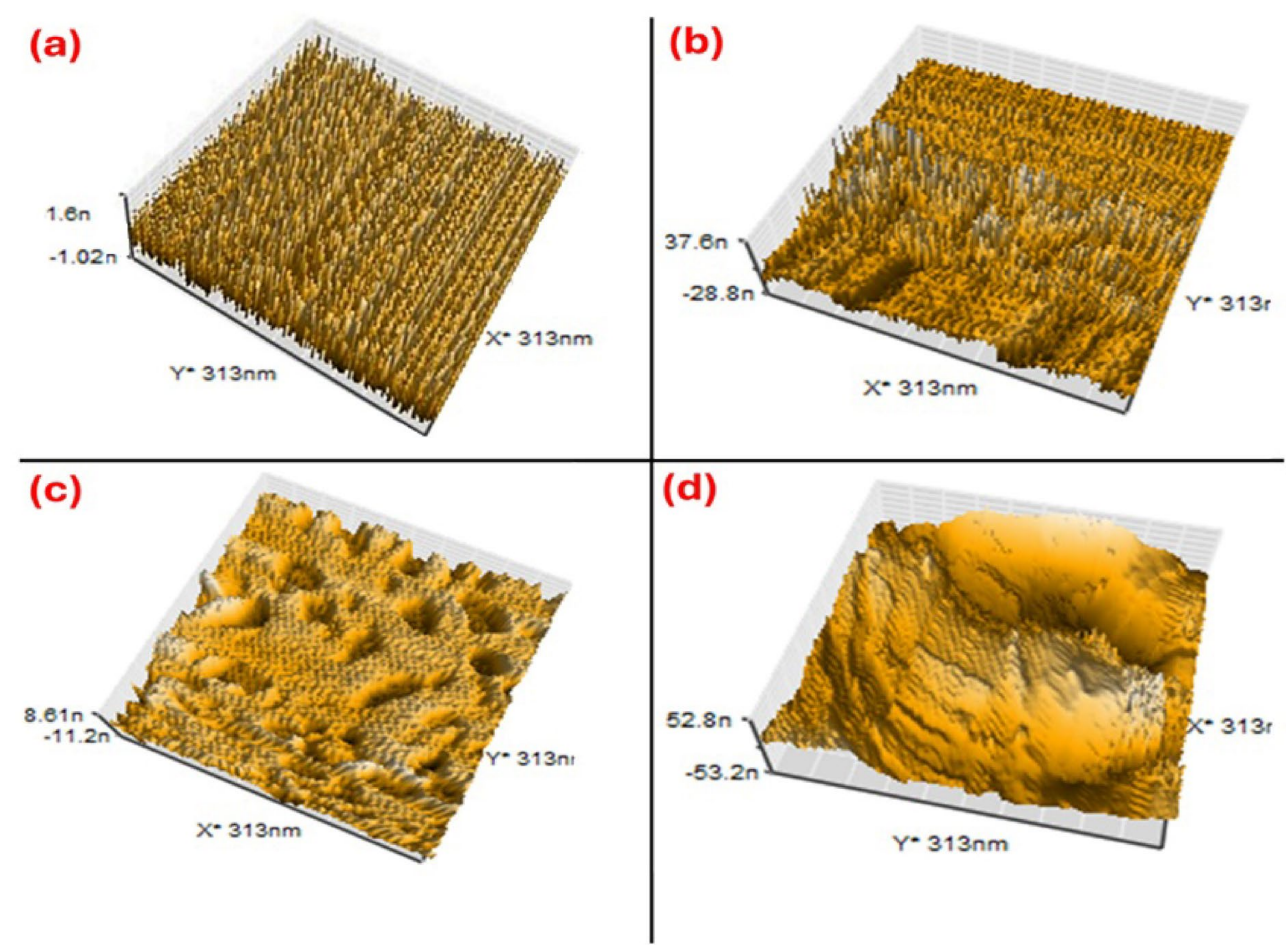
3D topographic AFM of GO, FG, G-TBAB, and Li-MWCNTs.

Atomic force microscopy (AFM) images acquired over a 313 nm × 313 nm scan area provide detailed insight into the surface morphology of GO and its chemically modified derivatives: FG, G-TBAB, and Li-MWCNTs. As shown in Fig. 7a, GO exhibits a relatively smooth and homogeneous surface, with a narrow height distribution ranging from approximately − 1.02 nm to 1.6 nm. This low roughness is characteristic of GO and reflects its layered morphology, decorated with oxygen-containing functional groups that promote a relatively flat surface profile^43^. In contrast, FG (Fig. 7b) exhibits a pronounced increase in surface roughness, with height variations spanning from − 28.8 nm to 37.6 nm over the same scan area. This pronounced roughness indicates that fluorine functionalization introduces surface defects and enhances vertical irregularity, likely due to lattice distortion and local disruption of the graphene sheets. Such features may also arise from partial exfoliation and wrinkling induced during the fluorination process^43,44^. The AFM image of G-TBAB (Fig. 7c) reveals a heterogeneous, irregular surface with distinct protrusions and depressions, with height variations ranging from − 11.2 nm to 8.61 nm. This morphology can be attributed to the attachment of bulky tetrabutylammonium bromide moieties, which hinder graphene restacking and lead to uneven layer aggregation. The observed surface heterogeneity is consistent with previous reports on graphene functionalized with large organic cations^45,46^. Li-MWCNTs (Fig. 7d) exhibit the most pronounced surface roughness among the investigated samples, with height variations ranging from − 53.2 nm to 52.8 nm. This extreme roughness reflects the inherent tubular morphology of MWCNTs combined with the effect of lithium fluoride incorporation, which introduces significant surface corrugation and structural deformation. The large vertical variations suggest strong interfacial interactions between LiF species and the carbon framework, resulting in a complex and highly textured surface topology^47^. Overall, AFM analysis confirms that chemical functionalization markedly alters the surface morphology of GO-derived materials. GO exhibits the smoothest and most uniform surface, whereas FG displays enhanced roughness due to fluorine-induced lattice disruption. G-TBAB exhibits moderate roughness associated with bulky organic functional groups, whereas Li-MWCNTs show the highest surface roughness due to the combined effects of nanotube architecture and LiF doping. A quantitative summary of AFM-derived surface roughness parameters and morphological characteristics is provided in Table 3.

**Table 3:** AFM features, including surface morphology, height range, surface roughness, structural characteristics, surface texture, and chemical effects on morphology, for GO, FG, G-TBAB, and Li-MWCNTs.

Parameter	GO	FG	G-TBAB	Li-MWCNTs
Surface morphology	Smooth and homogeneous	Rough with increased irregularities	Moderately rough with protrusions	Highly irregular with prominent surface features
Height range (nm)	− 1.02 to 1.6	− 28.8 to 37.6	− 11.2 to 8.61	− 53.2 to 52.8
Surface roughness	Low	High	Moderate	Very High
Structural characteristics	A layered structure with oxygen groups	Disrupted surface due to fluorine attachment	Irregular surface due to bulky organic groups	Tubular structures causing large corrugations
Surface texture	Flat and uniform	Wrinkled with sharp peaks and valleys	Rough with small bumps	Complex topology with deep pits and peaks
Notable features	Smoothest surface	Sharp peaks and more profound valleys	Distinct protrusions and irregularity	Most dramatic roughness and tubular patterns

**Fig. 8:**
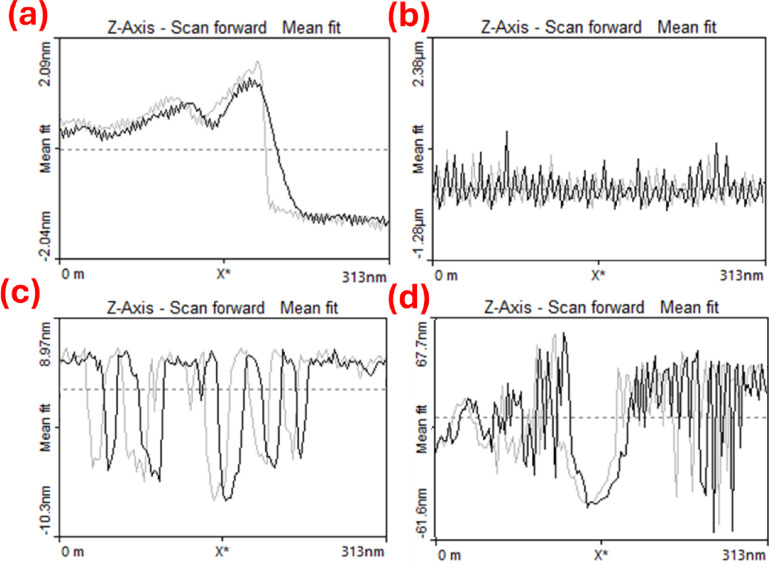
Roughness profiles of (**a**) GO, (**b**) FG, (**c**) G-TBAB, and (**d**) Li-MWCNTs. The curves highlight the differences in surface roughness among the prepared materials.

Figure 8 presents representative AFM roughness profiles extracted from the topographical images of GO, FG, G-TBAB, and Li-MWCNTs. The profiles reveal distinct surface-height variations following functionalization. GO and FG exhibited relatively small height fluctuations, indicating more homogeneous surface morphologies. In contrast, G-TBAB showed pronounced surface features associated with the introduction of TBAB species, while Li-MWCNTs displayed the largest height variations, reflecting the rough and heterogeneous structure induced by the incorporation of multi-walled carbon nanotubes. These results support the AFM observations and provide additional insight into the surface morphology and roughness evolution of the modified GO derivatives.

### Optical properties

#### UV–Vis spectroscopy

**Fig. 9:**
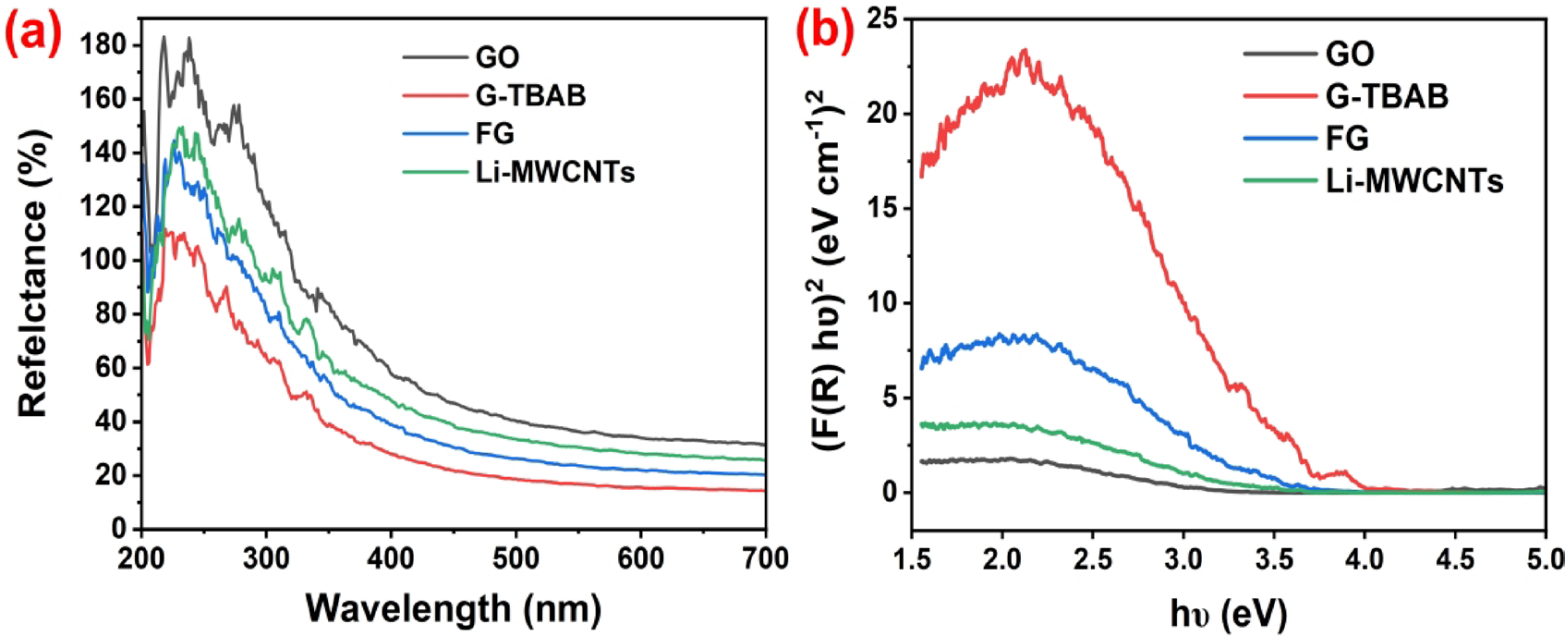
(**a**) UV–Vis spectra and (**b**) corresponding Tauc plots of GO, G-TBAB, FG, and Li-MWCNTs showing the band-gap evolution affected by chemical functionalization.

The UV–Vis diffuse reflectance spectra (Fig. 9a) exhibit a characteristic π–π* electronic transition across the wavelength range of 236–239 nm, indicative of the conjugated carbon framework in GO, G-TBAB, FG, and Li-MWCNTs. Corresponding Tauc plots (Fig. 9b) were constructed to estimate the optical band gaps (Eg), yielding values of 2.83 eV for GO, 3.72 eV for G-TBAB, 3.51 eV for FG, and 3.30 eV for Li-MWCNTs. The systematic increase in Eg following functionalization, particularly upon halogen incorporation, confirms effective modulation of the electronic structure through chemical doping. Such band gap widening is expected to influence charge-carrier dynamics and enable tunability of light-harvesting behavior, which is advantageous for optoelectronic and photocatalytic applications^48,49^. Comparable trends in band-gap enlargement have been reported for other doped carbon-based composite materials engineered for enhanced photocatalytic performance^17–20^.

#### Photoluminescence (PL) spectroscopy

**Fig. 10:**
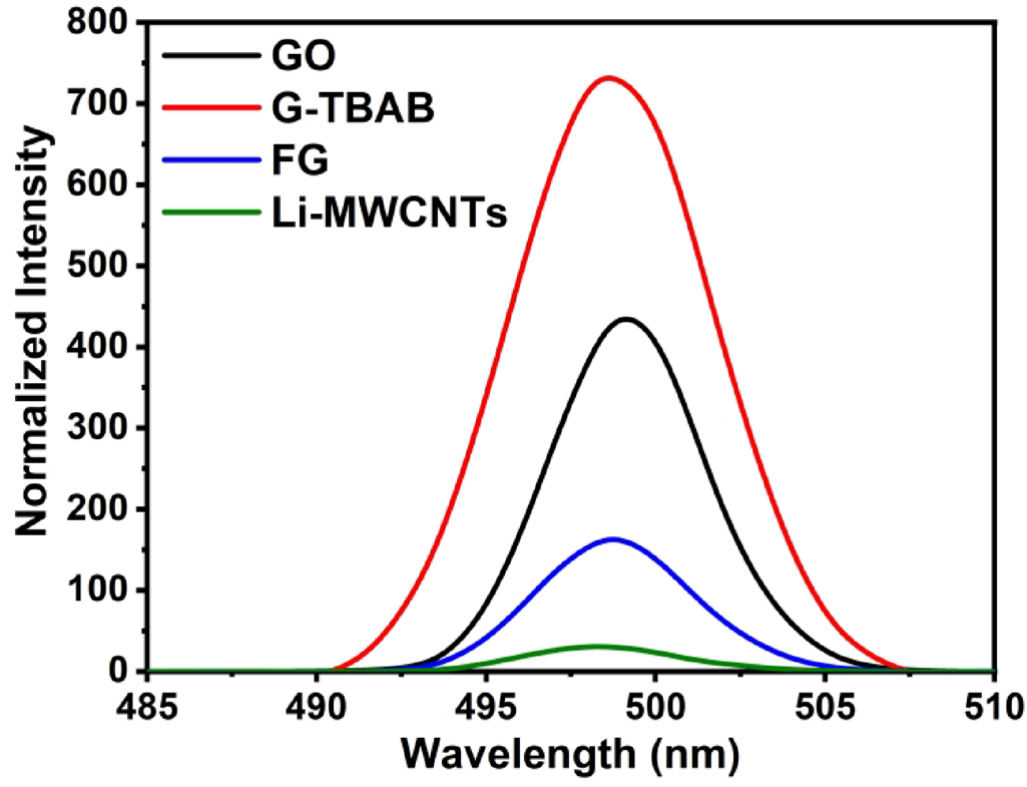
Photoluminescence (PL) spectra of GO, G-TBAB, FG, and Li-MWCNTs, illustrating the effect of functionalization on the optical emission behavior.

The photoluminescence (PL) spectra (Fig. 10) exhibit a prominent emission band centered at approximately 498 nm in GO, Li-MWCNTs, and FG, which is commonly attributed to charge recombination involving C–C and C–O electronic states. In contrast, G-TBAB exhibits a slight redshift to ~ 499 nm and shows the highest PL intensity among the samples, indicating enhanced radiative recombination efficiency. This behavior suggests increased defect- or functional-group-mediated emission pathways, rendering G-TBAB potentially attractive for light-emitting and optoelectronic applications. Conversely, Li-MWCNTs exhibited the lowest PL intensity, indicating effective suppression of charge-carrier recombination and prolonged carrier lifetimes. Such characteristics are advantageous for applications requiring efficient charge separation, including photovoltaic systems and energy storage materials^50^. Although these PL trends provide valuable insights into the electronic and recombination behavior of the materials, further device-level investigations are necessary to fully assess their practical performance. This observation is consistent with previous reports on halogen-doped carbon nanomaterials, where high PL intensity has been correlated with enhanced radiative recombination rates^51,52^. However, high PL intensity alone does not guarantee optimal performance in optoelectronic devices; other factors, such as charge-extraction efficiency, interfacial defects, and device architecture, also play critical roles. Therefore, while G-TBAB shows promise for fluorescence-based sensors or luminescent applications where strong emission is desirable, further device-level studies are required to confirm its practical efficacy. The high PL intensity also suggests fewer non-radiative recombination pathways, minimizing energy loss as heat, which could be beneficial for luminescent materials and display technologies, though this remains to be validated in functional devices. In contrast, the GO and FG samples exhibit intermediate PL intensity, which might be suitable for optical sensors under specific conditions^53^. Their moderate PL intensity indicates some degree of charge separation, which has been suggested to be beneficial for hybrid optoelectronic devices such as solar cells or photosensors in prior literature^50,54^, but direct evidence in the present system requires further investigation. On the other hand, the Li-MWCNTs sample shows the lowest PL intensity, indicating slower recombination and efficient charge separation. This property could enhance the charge storage capacity, conductivity, and electrochemical stability of the material, potentially making it a candidate for high-performance supercapacitors and advanced battery materials, as supported by similar trends observed in lithium-functionalized carbon systems^55,56^. However, its low PL intensity suggests it may be less effective for optical emission-based applications^50^, though this does not preclude its use in other optoelectronic contexts such as charge-transport layers.

### Electrochemical impedance spectroscopy (EIS) analysis

**Fig. 11:**
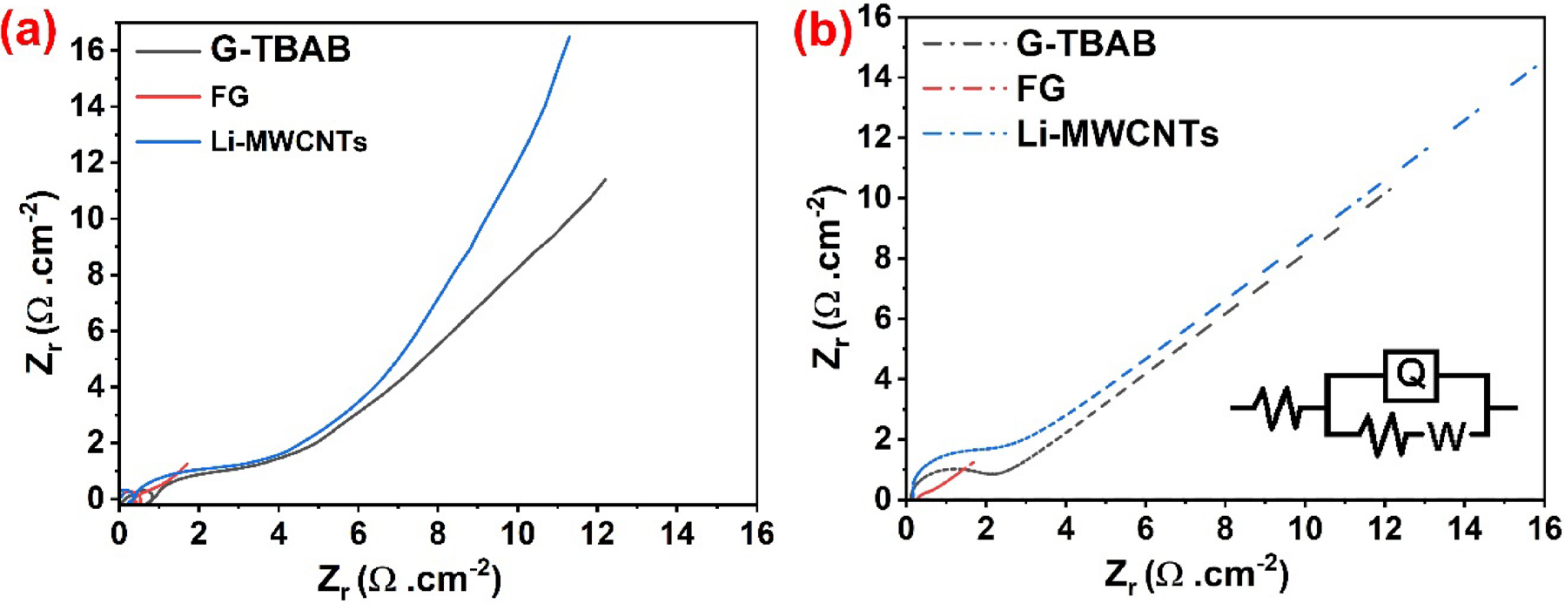
Resistance detection of G-TBAB, FG, and Li-MWNCTs films between ITO and Au as transparent conductive electrode (TCE) and counter electrode (CE) using EIS (**a**) and its integrated EEC (**b**) reveals differences in charge-transfer resistance and ion diffusion for different prepared carbon-based derivatives.

EIS Nyquist plots and their corresponding fitted equivalent electrical circuit (EEC) models (Fig. 11a and b) provide detailed insight into the charge transport and interfacial processes within the fabricated films. The selected equivalent circuit consists of R_f_, R_CT_, QC, and W. This model was chosen because it adequately captures the combined effects of interfacial charge transfer, non-ideal capacitive behavior, and ion diffusion within the modified GO-derivative films, as evidenced by the excellent agreement between the experimental and fitted EIS spectra. The R_f_ represents the intrinsic resistance of the electrolyte, substrate, and electrical contacts, reflecting the overall ohmic losses in the system. The R_CT_ describes the resistance to electron transfer across the electrode/electrolyte interface, where lower values indicate more efficient charge-transfer kinetics. The QC accounts for the non-ideal capacitive behavior arising from surface roughness, structural heterogeneity, and distributed active sites. The exponent *n* of the constant phase element QC describes the deviation from ideal capacitive behavior, where *n* = 1 corresponds to an ideal capacitor and decreasing values indicate increasing surface heterogeneity and non-ideal interfacial behavior^57,58^. The W element represents diffusion-controlled charge transport and provides information on ion mobility and mass-transfer limitations. The employed circuit model is shown as an inset, and the extracted electrochemical parameters are summarized in Table 4. Among the investigated materials, FG exhibits the lowest series sheet resistance (R_f_ = 1.19 Ω·cm^−2^) and charge transfer resistance (R_CT_ = 2.54 Ω·cm^−2^), indicating highly efficient charge transport across the electrode–electrolyte interface. This superior performance can be attributed to fluorine-induced electronic modulation, where the high electronegativity of fluorine promotes charge delocalization, suppresses recombination losses, and enhances electrical conductivity^59–62^. In Addition, FG displays the lowest interfacial capacitance (QC), suggesting reduced charge accumulation and rapid carrier extraction. Collectively, these characteristics make FG particularly suitable as a charge-transport layer or counter-electrode material for photovoltaic and electrocatalytic devices. In contrast, G-TBAB exhibits a higher R_CT_ value (12.91 Ω cm^−2^), indicating increased resistance to interfacial charge transfer. The incorporation of bulky TBAB groups likely introduces steric hindrance, which limits charge mobility across the graphene surface. Nevertheless, the moderate values of QC and R_f_ indicate a reasonable balance between charge transport and charge storage, consistent with other quaternary ammonium salt (QAS)- modified carbon systems reported for hybrid energy-harvesting and storage devices^63–66^. Li-MWCNTs exhibited the highest resistance values among all samples, with R_f_ = 1.70 Ω cm^2^ and R_CT_ = 17.73 Ω cm^−2^, implying limited electrical conductivity and sluggish charge transfer. The incorporation of LiF may introduce structural defects or charge-trapping sites that impede electronic transport. However, Li-MWCNTs exhibit the highest interfacial capacitance (QC = 7.83 × 10–4 F.cm^−2^) along with a pronounced W impedance (3.97 × 10^−2^ Ω.cm^−2^), indicative of enhanced ion diffusion and adsorption/insertion processes. This electrochemical signature is characteristic of materials optimized for energy storage applications, particularly supercapacitors and battery electrodes^24,37,67^.

**Table 4:** Key electrochemical parameters from the fitted EIS of G-TBAB, FG, and Li-MWCNTs as films on ITO.

Parameters	R_f_ (Ω.cm^−2^)	QC (F.cm^−2^)	n	R_CT (_Ω.cm^−2^)	W (Ω.cm^−2^)	R_SE_ (%)
G-TBAB	1.51	1.76E-04	0.49	12.91	5.17E-02	7.935
FG	1.19	1.10E-04	0.53	2.54	3.25E-02	3.841
Li-MWCNTs	1.70	7.83E-04	0.48	17.73	3.97E-02	7.369

Overall, the EIS analysis clearly differentiates the functional roles of the synthesized materials. FG demonstrates outstanding charge-transfer properties (R_CT_ = 2.54 Ω·cm^2^), making it the most promising candidate for photovoltaic and electrocatalytic applications. G-TBAB exhibits intermediate behavior, suitable for hybrid optoelectronic systems that require a balance between transport and storage. Li-MWCNTs, despite their higher resistances, show strong ion diffusion and capacitive characteristics, highlighting their potential for energy storage technologies such as supercapacitors and batteries^62,67^.

### Comprehensive comparison

This study lists the performance data of the self-synthesized carbon-based derivatives in Table 5. When compared with data from representative recent literature, differences in synthesis and testing conditions prevent direct quantitative comparison, but the data obtained in this work align with previously reported trends, which verifies that the three types of materials are suited for photovoltaic, charge transport, and energy storage application scenarios, respectively.

**Table 5:** Comparison of the optical and electrochemical properties of the prepared carbon-based materials with representative literature, highlighting their potential applications.

Material	Optical properties	Electrical/ Electrochemical properties	Potential application	Main finding	Ref.
FG	Eg = 3.51 eV, PL peak = 498 nm	R_CT_ = 2.54 Ω·cm^2^	Photovoltaics, optoelectronic devices	Lowest charge transfer resistance and efficient charge transport	This work
Fluorinated graphene	3.1–3.8	High electrical conductivity	Solar cells, Optoelectronics	Suppressed charge recombination	^68^
Fluorinated graphene	Tunable band gap (1.82–2.99 eV)	Electronic structure modified by C–F bonds	Optoelectronic, photonic devices	Fluorination enables band-gap engineering	^16^
Fluorine-doped CNTs	Modified electronic structure	Enhanced electron transfer and catalytic activity	Catalysis, electrochemical systems	F doping increases the positive charge on carbon atoms	^69^
GO-TBAB	Eg = 3.30 eV, highest PL intensity	R_CT_ = 12.91 Ω·cm^2^	Energy harvesting, sensors	Balanced charge transport and charge storage	This work
Graphene/TBAB	Stable carrier modulation	Conductivity increased from 5.08 × 10^6^ to 3.77 × 10^7^ S·m^−1^	Electronic devices, transparent electrodes	TBAB efficiently tunes carrier concentration and conductivity	^70^
Quaternary ammonium-functionalized GO (GO/CTAB)	Improved interfacial interaction	Improved GO dispersion and electrical performance	Polymer nanocomposites, conductive composites	Quaternary ammonium salts enhance GO compatibility	^71^
Li-MWCNTs	Eg = 3.72 eV, PL peak = 498 nm	R_CT_ = 17.73 Ω·cm^2^	Energy storage	Highest ion-storage capability among the prepared samples	This work
Li-functionalized MWCNTs	Improved structural stability	Enhanced ion diffusion and hydrogen storage	Hydrogen storage, energy storage	Li functionalization improves adsorption and electrochemical behavior	^72^
LiF/MWCNT composite	–	Capacity retention 70.1% after 200 cycles; 274 mAh g⁻^1^	Lithium-ion batteries	LiF stabilizes the SEI layer and improves electrochemical performance	^37^

## Conclusions

This study presents a comprehensive synthesis and characterization of hetero-functionalized carbon allotropes: bromine-functionalized graphene (G-TBAB), fluorine-functionalized graphene (FG), and lithium-functionalized multi-walled carbon nanotubes (Li-MWCNTs). FTIR and XPS confirmed the successful attachment of TBAB (N–H, C–N), fluorine (C–F), and lithium (Li–O) functionalities, with the dopant’s reducing influence on the GO sheet, as evidenced by increased C/O ratios. XRD analysis revealed structural modifications, including the disappearance of the GO peak at ~ 10° and the emergence of new peaks corresponding to functionalized structures. HR-TEM showed distinct morphologies: FG presented rigid, layered sheets; G-TBAB showed dispersed flakes with reduced aggregation; Li-MWCNTs maintained tubular structures with localized surface roughness. AFM analysis revealed that FG exhibits higher surface roughness compared to other carbon derivatives, whereas G-TBAB shows intermediate roughness. PL spectroscopy revealed that Br-GO shows the highest emission intensity, indicating rapid radiative recombination, while Li-MWCNTs show the lowest intensity, suggesting slower recombination. EIS results demonstrate that FG is the most conductive among the three materials under the tested conditions, whereas G-TBAB and Li-MWCNTs exhibit different electrochemical signatures. This work establishes structure–property relationships for three functionalized carbon allotropes. The data show that fluorine functionalization correlates with reduced charge-transfer resistance, bromine functionalization with increased photoluminescence, and lithium functionalization with increased capacitance.

## Data Availability

All data supporting the findings of this study are available within the paper and its Supplementary Information.

## Abbreviations

AFM: Atomic force microscopy
AC: Alternating current
Au: Gold
Br: Bromine
CPE: Constant phase element
CNTs: Carbon nanotubes
DI: Deionized water
EEC: Equivalent electrical circuit
EDX: Energy-dispersive X-ray spectroscopy
Eg: Optical band gap
EIS: Electrochemical impedance spectroscopy
FESEM: Field-emission scanning electron microscopy
FG: Fluorinated graphene
FTIR: Fourier-transform infrared spectroscopy
GO: Graphene oxide
G-TBAB: Tetrabutylammonium bromide–functionalized graphene
HF: Hydrofluoric acid
HR-TEM: High-resolution transmission electron microscopy
ITO: Indium tin oxide
LED: Light-emitting diode
Li: Lithium
LiF: Lithium fluoride
Li-MWCNTs: Lithium-functionalized multi-walled carbon nanotubes
MWCNTs: Multi-walled carbon nanotubes
PL: Photoluminescence
QAS: Quaternary ammonium salts
Qc: Interfacial capacitance
Rf: Film resistance
RSS: Series sheet resistance
Rct: Charge transfer resistance
SEM: Scanning electron microscopy
sp^2^ / sp^3^: Sp^2^ / sp^3^ hybridized carbon
TBAB: Tetrabutylammonium bromide
TEM: Transmission electron microscopy
UV–Vis: Ultraviolet–visible spectroscopy
W: Warburg impedance
XPS: X-ray photoelectron spectroscopy
XRD: X-ray diffraction
